# Supplementary material for: The Activation of the Fibrodysplasia Ossificans Progressiva-Inducing ALK2-R206H Mutant Depends on the Distinct Homo-Oligomerization Patterns of ACVR2B and ACVR2A
Source: Cells. 2024 Jan 25;13(3):221. doi: 10.3390/cells13030221 (PMC10854824; doi:10.3390/cells13030221)
Supplement: Supplementary file 1 [file cells-13-00221-s001.zip › cells-2807760-supplementary.pdf]

SUPPLEMENTARY INFORMATION

Supplementary Figures S1 – S7

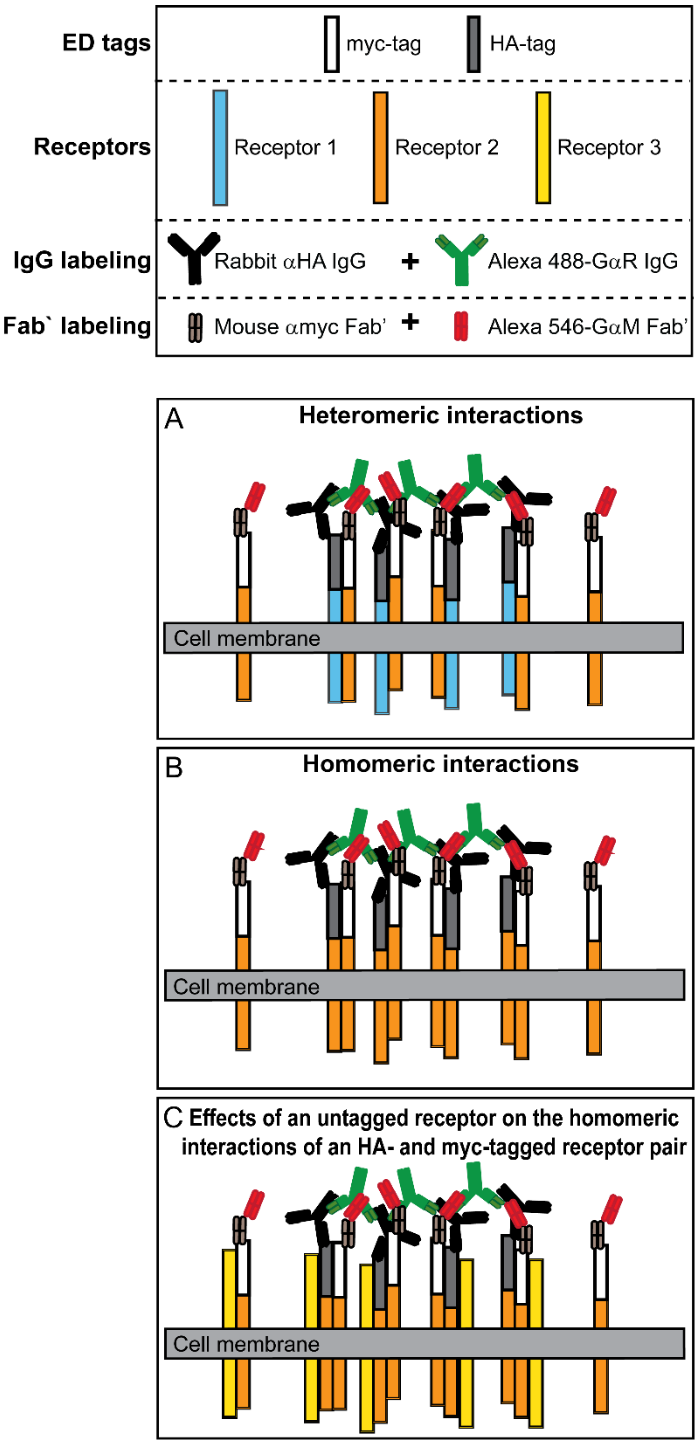

Figure S1. Schematic illustration of various combinations of patch/FRAP studies. Two receptors (receptor 1 and receptor 2) carrying different extracellular epitope tags (here, myc and HA) are coexpressed at the cell surface. They can be different receptors (to measure heteromeric interactions) or differently-tagged variants of the same receptor (for homomeric interactions). One tagged receptor (in the current study, the HA-tagged receptor) is immobilized by crosslinking with a double layer of IgGs (primary rabbit αHA IgG, and

secondary Alexa 488-GαR IgG with green fluorescence. The coexpressed myc-tagged receptor 2 is labeled by monovalent murine αmyc Fab' followed by Alexa 546-GαM Fab' (red fluorescence) secondary antibody. The effect of IgG crosslinking of HA-receptor 1 on the lateral diffusion of the Fab'-labeled receptor 2 is measured by FRAP. As explained under Results, the effect of immobilizing receptor 1 on the diffusion of receptor 2 can be either a reduction in its mobile fraction (in the case of stable interactions on the FRAP timescale), or a reduction in the lateral diffusion rate (in the case of complexes which are transient on that scale). To measure the effect of another receptor (untagged receptor 3) on the interactions between the HA- and myc-tagged receptors, receptor 3 is coexpressed with the tagged receptors. (**A**) Measurement of heteromeric interactions. The effect of IgG crosslinking of the HA-tagged receptor (*e.g.*, HA-ALK2-WT) on the lateral diffusion of a coexpressed, Fab'-labeled myc-tagged receptor (*e.g.*, myc-ACVR2A) is measured. (**B**) Homomeric interactions. Similar to panel A, except that the HA- and myc-tagged receptors are variants of the same receptor (*e.g.*, HA-ALK2-WT and myc-ALK2-WT). (**C**) Effect of a third untagged receptor on the homomeric interactions between HA- and myc-tagged pair of receptors. Here, a third untagged receptor (e.g., untagged ACVR2A) is coexpressed together with HA- and myc-tagged versions of the same receptor (e.g., HA-ALK2-WT and myc-ALK2-WT); the HA-tagged receptor is crosslinked by IgG, and the effects on the lateral diffusion of the Fab'-labeled myc-tagged receptor are measured by FRAP. Moreover, incubation with ligand during the patch/FRAP experiment can be used to test the effect of ligand binding on the interactions measured.

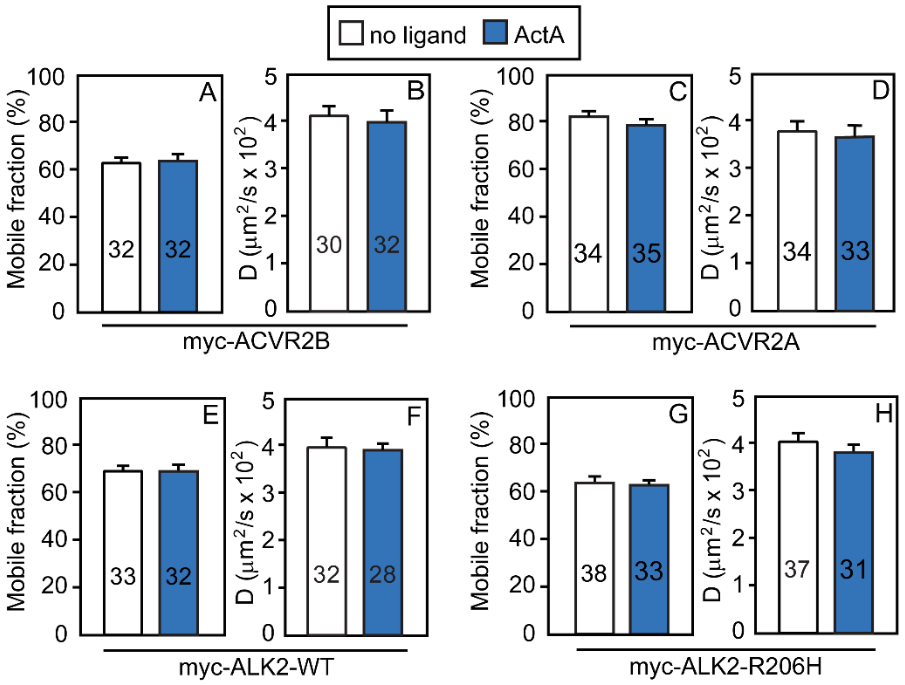

Figure S2. ActA does not alter the lateral diffusion of singly expressed ALK2-WT, ALK2-R206H, ACVR2A, or ACVR2B. FRAP experiments were as in Figure 1, except that no IgG crosslinking was applied. Cells were transfected with expression vectors encoding myc-tagged ACVR2B, ACVR2A, ALK2-WT or ALK2-R206H. After 24 h, live cells were subjected to fluorescent antibody labeling by monovalent Fab' fragments, and were taken for FRAP measurements. (**A, C, E, G**) Average R_f values; (**B, D, F, H**) average D values. The bars depict the average values (mean ± SEM); the number of measurements (each conducted on a different cell) is shown on each bar. No significant differences were found between the R_f or D values of the pairs within each panel (n.s.= not significant; Student's two-tailed *t*-test).

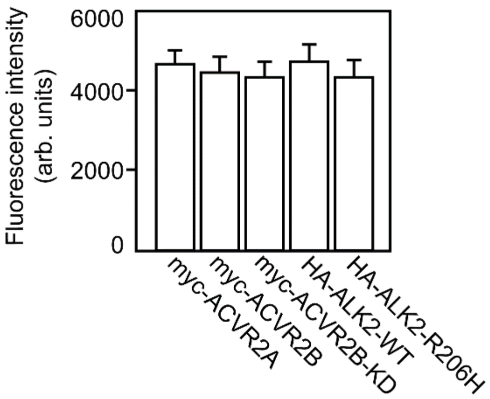

Figure S3. The cell-surface levels of epitope tagged receptors expressed in COS7 cells for the patch/FRAP studies are similar. COS7 cells were transfected by the indicated myc-or HA-tagged receptors. At 24 h post-transfection, the receptors were labeled at the cell surface in the cold (4 °C) by murine Fab' αHA or αmyc followed by secondary fluorescent Fab' GαM, and their levels were measured by the point confocal method as described in Figure 3A. Results in each bar are mean ± SEM of 30 independent measurements, each on a different cell. No significant differences were detected between the expression levels of the receptors (one-way ANOVA and Bonferroni post-hoc test).

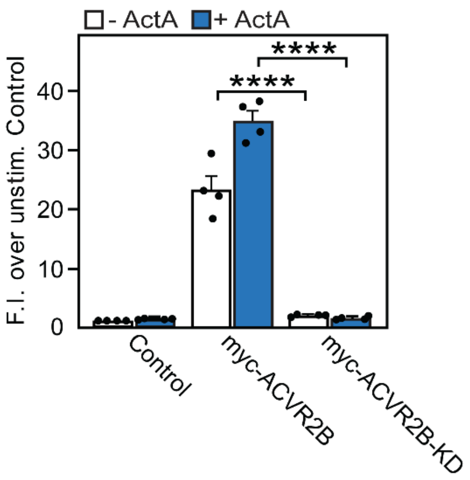

Figure S4. Kinase-dead myc-ACVR2B-K217R does not confer ActA-mediated transcriptional activation of BRE-Luc. U2OS cells were transfected with BRE-Luc and pRL-TK together with empty vector (control), myc-ACVR2B or myc-ACVR2B-KD. At 17 h post-transfection, the cells were serum-starved (5 h) and stimulated (or not) with ActA (2 nM, 19 h). Relative Luminescence Units (RLU) are expressed as mean fold induction ± SEM (n = 4 independent experiments). Renilla luminescence in the DLR luminescence assay served to normalize for transfection efficiency. The value obtained for untreated and unstimulated cells was taken as 1. The cell-surface levels of the tagged receptors were not altered by the co-expressed receptors (Figure S7). Asterisks show significant differences between the pairs indicated by the brackets, using one-way ANOVA and Bonferroni post hoc test (****, p < 10-4). While expression of the wild-type myc-ACVR2B led to significant activation of the BRE-Luc, which increased in the presence of ActA, expression of myc-ACVR2B-KD did not induce BRE-Luc transcription either in the absence or presence of ActA.

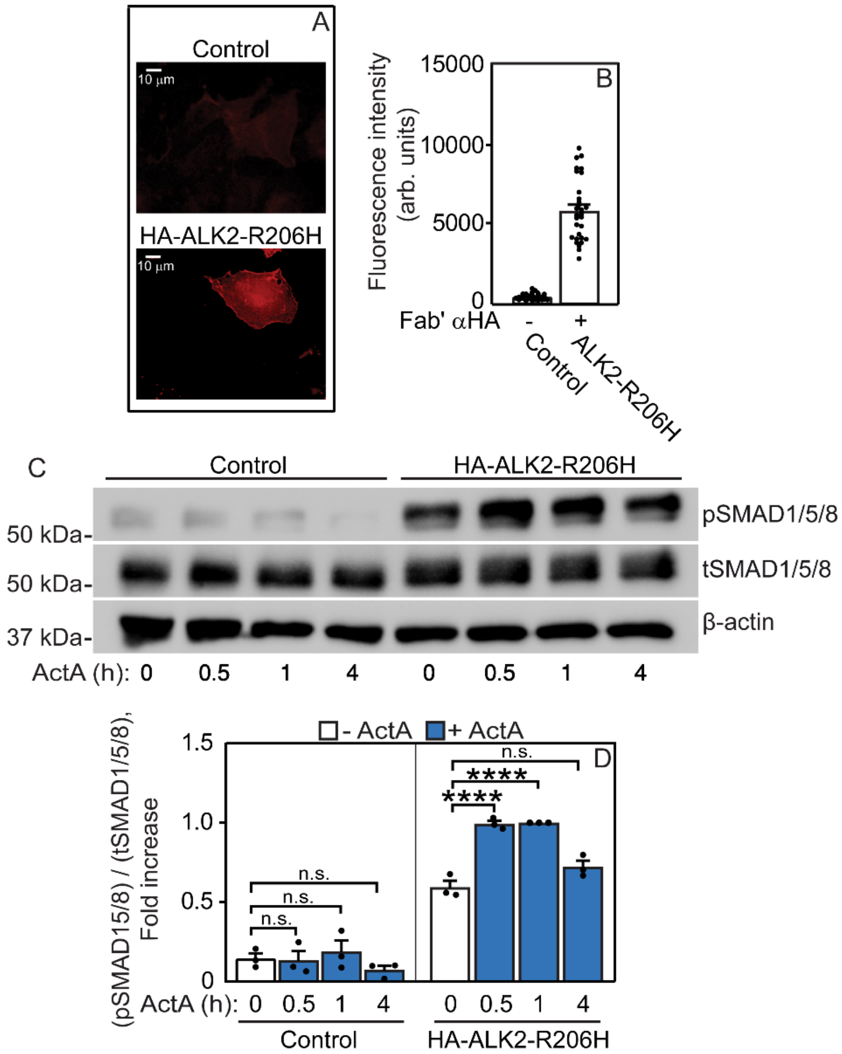

Figure S5. SMAD1/5/8 activation by ActA as a function of time in control and ALK2-R206H transfected U2OS cells. Experiments were conducted as in Figure 7, except that the cells were transfected with HA-ALK2-R206H (or empty vector; control). At 24 h post-transfection, the cells were starved (2 h, 1% FCS) and activated (or not; time 0) with ActA (4 nM, 37 °C) for the indicated times. This was followed by lysis and immunoblotting for pSMAD1/5/8, tSMAD1/5/8, and β-actin as in Figure 7. (**A**) Typical fluorescent images of cells subjected to measurements of the cell-surface levels of HA-ALK2-R206H to validate transfection. Scale bars, 10 µm. Labeling was with a saturating concentration (40 µg/ml) of murine Fab' αHA followed by 40 µg/ml Alexa 546-Fab' GαM. Micrographs were obtained using Zeiss Axioimager.D1 microscope with a 63x oil-immersion objective. (**B**) Quantification of the cell surface levels of HA-ALK2-R206H using the point-confocal method, as described in Figure 3A. Data are mean ± SEM of 30 measurements under each condition. (**C**) Representative blots of the time dependence of SMAD1/5/8 phosphorylation by ActA. (**D**) Quantification of ActA-mediated pSMAD1/5/8 formation. The bands were visualized by ECL and quantified by densitometry (Materials and Methods). Data are mean ± SEM (3 independent experiments in each case), presented as the ratio of pSMAD1/5/8 over tSMAD1/5/8. The values obtained for 60 min stimulation of cells transfected with HA-ALK2-R206H were taken as 1. Asterisks indicate significant differences between the bracketed pairs (one-way ANOVA and Bonferroni post hoc test; ****, $p < 10^{-4}$; n.s. = not significant).

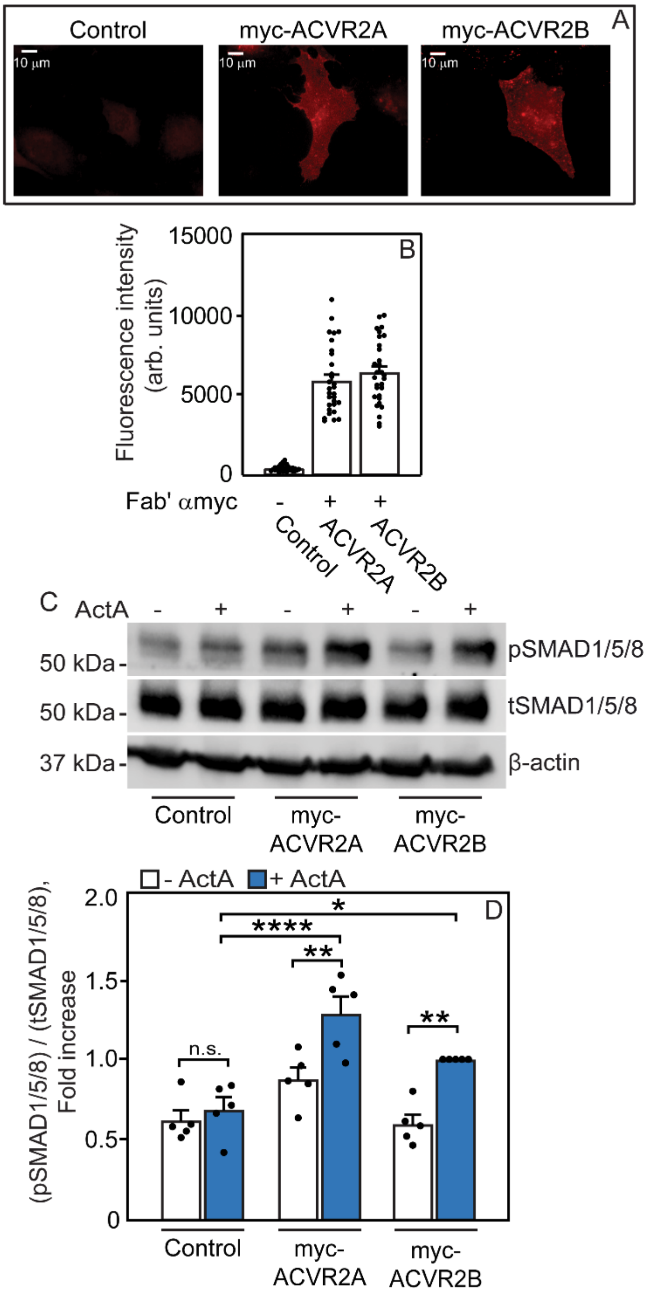

Figure S6. Signaling activity of myc-ACVR2A and myc-ACVR2B. U2OS cells were transfected with myc-ACVR2A, myc-ACVR2B or empty vector (control). After 24 h, cells were starved (2 h, 1% serum) and stimulated (4 nM ActA, 60 min, 37 °C). They were then lysed and tested by immunoblotting for pSMAD1/5/8, tSMAD1/5/8 and β-actin. (**A**) Representative fluorescence images of cells expressing myc-tagged ACVR2A or ACVR2B, to validate transfection. Scale bars, 10 µm. Labeling and micrography were as in Figure S2, except that the primary antibody was murine Fab' αmyc. (**B**) Quantification of the cell surface levels of myc-tagged ACVR2A or ACVR2B by the point-confocal method, as described in Figure 3A. Data are mean ± SEM of 30 measurements under each condition. (**C**) Representative blots of SMAD1/5/8 phosphorylation. (**D**) Quantification of ActA signaling to SMAD1/5/8 by myc-ACVR2A or myc-ACVR2B. The bands were quantified by ECL and densitometry. Data are mean ± SEM of the pSMAD1/5/8 over tSMAD1/5/8 ratio of 5

independent experiments. The value obtained for ActA-stimulated cells transfected with myc-ACVR2B was taken as 1. A significant increase in the signaling was observed following transfection with either of the myc-tagged constructs (one-way ANOVA and Bonferroni post-hoc test; *, $p < 0.04$; **, $p < 3 \times 10^{-3}$; ****, $p < 10^{-4}$).

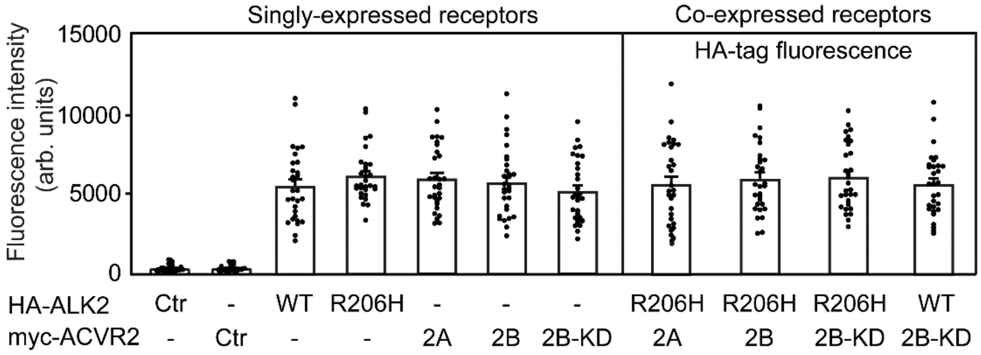

Figure S7. The cell-surface levels of the tagged receptors transfected in the signaling studies are similar, and are not affected by co-expression of myc-tagged ACVR2A/B. U2OS cells were singly transfected by HA-tagged ALK2-WT or ALK2-R206H, or by myc-tagged ACVR2A, ACVR2B or ACVR2B-KD (left panel). Where indicated (right panel), HA-tagged ALK2-R206H or ALK2-WT were co-expressed with a myc-tagged ACVR2 variant. After 24 h, the cell surface epitope-tagged receptors were labeled by murine Fab' αHA or αmyc followed by secondary fluorescent Fab' GαM, and their levels were measured by the point confocal method as described in Figure 3A and Figure S2. Results are mean ± SEM of 30 independent measurements (each on a different cell) under each condition. No significant differences were found between the expression levels of the singly-expressed receptors, and the levels of HA-tagged ALK2-R206H or ALK2-WT were not altered by co-expression with myc-ACVR2A/B or myc-ACVR2B-KD (one-way ANOVA and Bonferroni post-hoc test).
